# Herbal Ingredients in the Prevention of Breast Cancer: Comprehensive Review of Potential Molecular Targets and Role of Natural Products

**DOI:** 10.1155/2022/6044640

**Published:** 2022-08-16

**Authors:** Esra Küpeli Akkol, Hilal Bardakci, Timur Hakan Barak, Michael Aschner, Gökçe Şeker Karatoprak, Haroon Khan, Yaseen Hussain

**Affiliations:** ^1^Department of Pharmacognosy, Faculty of Pharmacy, Gazi University, Etiler, 06330 Ankara, Turkey; ^2^Department of Pharmacognosy, Faculty of Pharmacy, Acibadem Mehmet Ali Aydınlar University, 34752 Istanbul, Turkey; ^3^Department of Molecular Pharmacology, Albert Einstein College of Medicine, Bronx, NY 10463, USA; ^4^Department of Pharmacognosy, Faculty of Pharmacy, Erciyes University, 38039 Kayseri, Turkey; ^5^Department of Pharmacy, Abdul Wali Khan University Mardan, 23200 Mardan, Pakistan; ^6^College of Pharmaceutical Sciences, Soochow University, Suzhou, Jiangsu, China

## Abstract

Among various cancers, breast cancer is the most prevalent type in women throughout the world. Breast cancer treatment is challenging due to complex nature of the etiology of disease. Cell division cycle alterations are often encountered in a variety of cancer types including breast cancer. Common treatments include chemotherapy, surgery, radiotherapy, and hormonal therapy; however, adverse effects and multidrug resistance lead to complications and noncompliance. Accordingly, there is an increasing demand for natural products from medicinal plants and foods. This review summarizes molecular mechanisms of signaling pathways in breast cancer and identifies mechanisms by which natural compounds may exert their efficacy in the treatment of breast cancer.

## 1. Introduction

Cancer is a critical health condition around the world. Breast cancer is the second most prevalent type of cancer and the main cause of death in women [1–3]. Tissue homeostasis is regulated by the normal functioning of cell division and death. The upregulation in these physiological mechanisms leads to cancer formation [4, 5]. Various intrinsic and extrinsic elements might prompt breast cancer and exacerbate the condition [6]. Adverse effects of common treatment strategies such as chemo- and radiotherapy adversely affect patients rendering treatment more challenging [7, 8]. In addition, multidrug resistance (MDR) is an additional obstacle for current conventional treatment strategies [9, 10]. Thus, original and alternative treatment regimens have been investigated [11–14].

A variety of pathways are involved in the pathogenesis of breast cancer and the proliferation of cancer cells [15–18]. As an example, genes encoding the units of cell cycle like cyclin and CDKs along with their intrinsic inhibitors are dysfunctional in most cancers [19, 20]. Overactivity of CDKs is generally due to either cyclin overexpression or intrinsic CDKI downregulation [21]. Thus, CDK inhibition is considered a promising approach to cancer therapy [22]. To identify novel treatment in breast cancer, researchers have focused on various pathways such as Wnt, Notch, and SHH [23–27]. Breast physiology involves extracellular matrix (ECM) and several stromal cells such as immune and endothelial cells, adipocytes, and fibroblasts [28]. The majority of cancer stroma consists of cancer-associated fibroblasts (CAFs) which endorse cancer arousal, angiogenesis, invasion, and metastasis [28]. Since CAFs also lead to drug resistance, targeting CAFs helps in managing breast cancer with MDR [28]. Survival of cancerous cells and their spread are improved by the expression of several Notch molecules and release factors [29–31]. Targeting transforming growth factor-*β* (TGF-*β*) signaling aids in tumor suppression and promotion [32]. Several natural compounds involve in the mentioned mechanisms and might be promising treatment strategies in breast cancer [33]. More than 50% of modern medicines have been developed from natural products and 75% of anticancer medicines are of natural sources [34]. Cancer is one of the rebellious diseases in which natural metabolites have been active via different mechanisms [35, 36]. Current researches verified the utilization of natural compounds in the prevention and treatment of breast cancer [37].

This review addresses pathways and mechanisms in breast cancer development. Moreover, natural compounds and their relationship to epigenetic modifications, aromatase activity, and arachidonic acid pathway as well as cell apoptosis pathway are highlighted [38].

## 2. Signaling Pathways and Hormones Associated in Breast Cancer Cell Cycle and Survival

### 2.1. Cyclin-Dependent Kinases (CDKs)

The division of a cell to form two daughter cells, also known as a cell cycle, depends on the success of four discrete phases that follow each other successfully: G1, S, G2, and M phases. During the M phase, mitosis is followed by cytokinesis, and G1 phase represents a gap between the mitosis and DNA replication phase (S phase) during which the cells are metabolically active and growing; following the S phase, cells continue to produce proteins (G2 phase) to prepare for mitosis. G0 phase represents a quiescent stage where the cells are metabolically active but not proliferative until induced by appropriate signals [39].

Cyclin-dependent kinases (CDKs) are threonine/serine kinases. Regulation of their activity is through cyclins and CDK inhibitors (CKIs). CDKs form complexes with cyclins to regulate the transition between cycle phases [40, 41]. Phosphorylation of the CDKs at a conserved threonine is also involved in the full activation of most CDKs by CDK activating kinase (CAK) [41]. A series of CDKs also regulate the kinase activity by turning off the activity upon binding with active cyclin-CDK complexes. Two families of CDK inhibitors are Cip/Kip and INK4 family members. Progression through the G1 and S phase is regulated by the first via inhibiting the complexes of CDK2, CDK4, and CDK6 with cyclins A, D, and E. On the other hand, INK4 family members are specific for complexes of cyclin D with CDK4 and CDK6. The only regulation includes the progression through the restriction point in G1 [40–42]. Even though multiple loci encode CDKs and cyclins in human cells, only a number of them are precisely associated with the cell cycle, four CDKs including three interphases (CDK2, CDK4, and CDK6) and a mitotic (CDK1, likewise known as cell division control protein 2 (CDC2)) along with ten cyclins belonging to four distinct classes (A-, B-, D-, and E-type cyclins) [43]. Transition through subsequent phases of the cell cycle is controlled by specific CDK-cyclin complexes; for example, the complexes of CDK4 and CDK6-D-type cyclins control the progression via the G1 restriction point. On the other hand, G1 to S transition is regulated by CDK2/cyclin E complexes. The CDK2 complexes with cyclin A are required for progression through the S phase. The CDC2/cyclin B complexes regulate the G2 to M transition [41].

Hyperphosphorylation and deactivation of retinoblastoma protein (pRb) are mediated by CDK4 and CDK6–cyclin D complexes. When hyperphosphorylated, pRb releases E2F to express genes crucial to progress to the S phase. Therefore, by decelerating the progression of the cell cycle to the S phase, hypophosphorylated pRb serves as a tumor suppressor. CDK inhibitors prevent hyperphosphorylation of the pRb. Consequently, cells are arrested in the G1 phase, resulting in an indirect antitumor effect [44].

To develop effective treatments against cancer, the basic principles and regulators of the cell cycle, particularly CDKs, have long been targeted. It has been revealed that various tumorigenic incidents drive proliferation by affecting the complexes of CDK4 or CDK6 in the G1 or S phase or and G2/M control regulated by CDK1 and CDK2 [45, 46]. A broad array of carcinomas, sarcomas, and hematological malignancies were found to be associated with increased expressions of cyclin or CDK or decreased endogenous levels of CDK modulators/inhibitors including INK4 or CIP/KIP as reviewed by Roskoski. Although early identified inhibitors of CDKs were shown to be insufficiently active and exhibited toxicity, recently, palbociclib along with ribociclib and abemaciclib, selective inhibitors of CDK4/6, are approved for the treatment of breast cancer [46, 47].

Cyclin D1 multiplication has been shown in breast cancers [48, 49]. Cyclin D3 and E1 overexpression along with the reduced expression of p27Kip1 (CDKI) has also been shown in human breast cancer [50]. Cyclin gene (CCN) alterations were described to be associated with breast cancer [51]. High levels of cyclin B1 expression has been reported to be associated with poor survival in breast cancer [52]. Overexpression of cyclin D1 in mammary cells in transgenic mice resulted in abnormal mammary cell proliferation in addition to the increase of mammary adenocarcinomas suggesting that overexpression of cyclin D1 can induce tumorigenic changes in mammary tissues and assumes an important oncogenic role in breast cancer [53]. Although cyclin D1 knockout mice were shown to be resistant to the formation of Erbb2 or Ras oncogene-induced breast cancers, cyclin D1 deficiency has not been shown to be protective against tumor formation induced by c-Myc or Wnt-1 [54].

### 2.2. Notch Signaling

#### 2.2.1. Notch Ligands and Receptors

The Notch pathway regulates interactions between cells. One cell carries a ligand that combines with the other receptor [55, 56]. During development and homeostasis, Notch signaling controls various cell fate decisions in different tissues such as cell cycle progression, differentiation, maintenance, and self-renewal of stem cells [57]. In mammals, four heterodimeric transmembrane Notch receptors (NOTCH1-4) are identified along with five transmembrane ligands (Jagged 1 (JAG1), JAG2, Delta-like 1 (DLL1), DLL3, and DLL4) belonging to the Delta–Serrate–Lag (DSL) family [57, 58].

Notch receptors are 300-350 kDa transmembrane heterodimers composed of an intracellular and extracellular ligand-binding domain [59, 60]. The intracellular domain consists of a transmembrane region and an intracellular portion that controls signaling upon receptor ligation. The extracellular domain involves 10–36 repeats of an epidermal growth factor- (EGF-) like sequence motif and 3 repeats of a Lin-12/Notch/Glp (LNG) sequence motif whereas the intracellular domains involve six to seven Ankyrin repeats and a PEST-containing region [59]. Notch receptors traffic from the endoplasmic reticulum to the Golgi all along maturation and transported to the cell surface where a furin-like protease accomplishes first proteolytic cleavage (S1) [59]. The extracellular portion of Notch receptors is important for ligand binding that contains multiple epidermal growth factor- (EGF-) like repeats. The extracellular domain contains a negative regulatory region (NRR) which contains LIN12-NOTCH repeats and the heterodimerization domain. NRR, which is located between the ligand-binding and transmembrane regions, ensures that Notch signaling is inactive when there is no interaction with DSL ligands [60].

The canonical DSL ligands are type 1 cell-surface proteins with multiple tandem EGF repeats in their extracellular domains, structurally similar to Notch receptors. To bind to Notch, the DSL ligands need the DSL and the flanking N-terminal (NT) domains as well as the first two EGF repeats [61, 62]. In vertebrates, two different kinds of Serrate-like ligands (Jagged1 and Jagged2) contain nearly twice the number of EGF repeats as Delta-like ligands. They also contain an extra cysteine rich region which is absent in Delta-like ligands [63]. Multiple lysine residues and a C-terminal PDZ (PSD-95/Dlg/ZO-1) ligand motif constitute the DSL ligands [63].

The canonical Notch signaling pathway is stimulated by direct cell to cell contact when a ligand is combined with the receptor presented by the neighboring cell. After the cleavage, the Notch intracellular domain (NICD) translocates to the nucleus. This reaction triggers a set of biochemical reactions that can affect gene expression including proteolytic cleavage on the receptor. The Notch protein cleavage at the S2 cleavage site is mediated by ADAM10 and ADAM17, and the *γ*-secretase enzyme complex mediates another proteolytic cleavage. As a result, the NICD is released from the plasma membrane and moves to the nucleus and penetrates to the nuclear membrane [55, 56, 58]. In the nucleus, downstream target genes such as Hes and Hey family clusters, cyclin D1, and c-Myc expressed simultaneously to the transcriptional activation complex between the NICD and the transcription factor CSL (mammalian CBF-1) [55, 56].

Posttranslational modification also regulates Notch signaling. For example, peptide-O-fucosyltransferase (POFUT1) and the Fringe GlcNAc transferases sequentially glycosylate Notch receptors. As a result of this modification, the affinity of Notch for Delta and Serrate/Jagged ligands is altered. Phosphorylation of Notch proteins by glycogen synthase kinase 3*β* (GSK3*β*) or ubiquitination by the E3 ubiquitin ligase FBXW7 also occurs [58].

#### 2.2.2. Notch Signaling and Tumorigenesis

Notch activity is associated with both oncogenic and tumor-suppressive functions [57]. The role of Notch signaling in malignancy was first described in a case of human T lymphoblastic leukemia (T-ALL). A gene highly homologous to the *Drosophila* gene NOTCH, which was later named NOTCH-1, was identified at an uncharacterized locus in a t(7;9)(q34;q34.3) chromosomal translocation from that case which results in truncated transcripts [64, 65]. Next, Notch-1 attracted attention because alteration of Notch-1 was posited to play a role in the pathogenesis of several T cell neoplasms [64, 65]. The observations were supported by finding that more than 50% of human T-ALLs contain activating mutations which consist of the extracellular heterodimerization domain and/or the C-terminal PEST domain of NOTCH1 [66]. Diverse types of cancers such as gastric, cervical, colorectal, hepatocellular, lung, ovarian, pancreatic, and prostate are accompanied by elevated expression of Notch-3 and have been associated with rapid malignant progression, poorer prognosis, abnormal differentiation, and metastasis [65]. Thus, the role of Notch signaling in cancer seems diverse, because it can either be an oncogenic or tumor suppressor [67].

High levels of JAG1, JAG2, and NOTCH1 as well as DLL4 expression were detected and linked to poor survival or nodal and distant metastasis in human breast cancer [68–71]. Notch activation mediated by Jag1 was also shown to induce epithelial to mesenchymal transition, suggesting that ligand-induced Notch activation promotes tumor growth and metastasis [72]. Increased Notch signaling followed by the accumulation of the intracellular domain of Notch1 was detected in a wide variety of human breast carcinomas [73]. Notch1 was also found to be associated with mammary carcinogenesis in mice [74]. In ErbB2-negative breast tumor cell lines, Notch3-mediated signaling was shown to play an important part in the proliferation [75]. Overexpression of Notch3 caused cell cycle arrest at the G0/G1 phase. The proliferation and colony formation rates of MDA-MB-231 cells were also inhibited. Overexpression of intracellular domain of Notch3 upregulated Cdh1 expression. As a result, p27Kip accumulated by accelerating Skp2 degradation, leading to cell cycle arrest at the G0/G1 phase [76]. Carcinogenic properties of DLL1 were shown in ER*α*+ luminal human breast cancer cell lines, thus leading the poor prognosis of the disease [77, 78].

#### 2.2.3. Notch Signaling and Cross-Talk

The oncogenic functions of the Notch pathway are dependent on its ability to cross-talk with other pathways [79]. Cross-talk between various signaling pathways and Notch signaling have been documented in breast cancer. For instance, cross-linked function of Notch and EGFR (epidermal growth factor receptor) signaling is highly associated with occurrence of breast cancer which was reported when Notch1 overexpression promoted cell growth accompanied by upregulated EGFR expression levels. Moreover, EGFR and Notch1 expression was decreased by an EGFR inhibitor [80]. Further, Notch-1 transcriptional activity was suppressed by overexpression of (EGFR)-2 (ErbB-2 or HER2/neu) protein (ErbB-2) in breast cancer cells, and inhibition of ErbB-2 using trastuzumab reactivated Notch-1 activity which might explain the low efficacy of trastuzumab in sensitive cells or the development of resistance [81]. Notch and ErbB receptors in primary DCIS samples and cell lines displayed cross-talk regardless of the status of the ErbB2 receptor [82].

Notch-1 activity is inhibited by estrogen. Combinational treatment of antiestrogens with Notch inhibitors might be an effective option in ERalpha (+) breast cancers [83]. In addition, Jagged1/Notch1 signaling pathway can be mediated by 17*β*-estradiol suggesting a cross-talk between Notch signaling and 17 beta-estradiol and angiogenesis [84].

A study was conducted to analyze epithelial-endothelial cross-talk. The study revealed that cancer-secreted extracellular matrix protein 1 (ECM1) induced Notch-mediated endothelial feedback and enhanced migration and invasion to promote cancer progression [85].

Notch-induced AKT activation in MCF10A cells is required for Notch-induced protection against apoptosis. This observation was corroborated by decrement in AKT signaling by Notch inhibition [86]. A significant interaction was detected between Notch1, pAKT, and NF-*κ*B expression in TNBC [87]. Cartilage Oligomeric Matrix Protein (COMP) controls the cancer stem cell population via increasing Notch3 and Jag-ged1 interaction which results in increased Notch3 signaling activation. COMP-dependent activation of Notch3 also leads to cross-talk between *β*-catenin and AKT pathways [88].

Coordinated Notch1 and Ras/MAPK hyperactivation in breast cancer patient specimens was found to be related to poor general survival which led to the identification of cooperation between Notch and Ras/MAPK pathways [79].

#### 2.2.4. Notch and Tumor-Initiating Cells

Cancer-initiating cells (CICs) or cancer stem cells (CSCs) have substantial capacity for self-renewal and an ability to lead to the formation of heterogeneous lineages of cancer cells involving in the tumor. CICs originate from stem, progenitor, or differentiated cells [89].

Notch signaling promotes the proliferation of early progenitor cells and self-renewal of mammary stem cells suggesting its role in carcinogenesis [90]. In ductal carcinoma *in situ* (DCIS), the inhibition of Notch signaling pathways reduced DCIS mammosphere forming efficiency. Notch signaling was found to be crucial in nonadherent culture for self-renewal and cell survival [91]. In a breast cancer cell line, exposure to hypoxic environment induced 66 kDa isoform of the SHC gene (p66Shc), which controls the expression of Notch-3. Mammary gland stem/progenitor cells' self-renewal, as well as hypoxia survival, was modulated by p66Shc/Notch-3 interplay through inducing both Notch-ligand Jagged-1 and carbonic anhydrase IX gene [92]. Sansone et al. investigated the interaction of IL-6 and mammary stem/progenitor cells, as elevated serum levels of IL-6 have been associated with poor outcome in breast cancer patients, with results supporting the role of IL-6 in promoting malignant features in Notch-3-expressing progenitor/stem cells from human ductal breast carcinoma and normal mammary gland. IL-6 treatment triggered Notch-3-dependent upregulation of the Notch ligand Jagged-1 and carbonic anhydrase IX gene. It also promoted mammospheres and MCF-7-derived spheroid growth as well as a hypoxia-resistant/invasive phenotype in MCF-7 cells and mammospheres [92]. The inhibition of Notch1 caused growth arrest and inhibition of epithelial to mesenchymal transition in breast cancer stem cells [93].

#### 2.2.5. Notch and Triple-Negative Breast Cancer

Breast cancer is classified into different subtypes based on the presence or absence of human epidermal growth factor receptor 2 (HER2/neu), estrogen receptors (ERs), and progesterone receptors (PRs). Triple-negative breast cancer (TNBC) lacks all three receptors that are generally targeted in therapies; for that reason, TNBC is considered the most resistant subtype [94]. Notch1 and Notch3 were associated with TNBC pathogenesis or etiology, malignant or aggressive phenotypes, while Notch4 levels are important for promoting mesenchymal signature and keeping prostemless signaling constant during tumor progression of TNBC. On the other hand, the role of Notch-2 was reported to remain ambiguous. The authors noted that there is more evidence supporting the tumor-suppressive role rather than an oncogenic role [94].

Notch was advanced as a target with potential therapeutic effects in 30% of solid-type adenoid cystic carcinoma (ACC) of breast cancers [95]. A small-molecule drug ABT-737 and tumor-suppressive microRNA (miRNA) miR-34a, which are promising candidates for TNBC therapy, were encapsulated in poly(lactic-co-glycolic acid) nanoparticles (NP) and functionalized with Notch-1. Codelivery of Notch-1 antibodies and ABT-737 or miR-34a mediated by NP was pointed as an effective treatment approach for TNBC [96, 97]. The doxorubicin chemosensitivity of wild-type and chemoresistant MB-231-MDA TNBC cell line was increased by silencing of genes including Notch-1 as well as STAT-3 and *β*-catenin [98]. A combination of an oral selective gamma-secretase (GS) inhibitor RO4929097 with neoadjuvant carboplatin and weekly paclitaxel was recently investigated in a Phase I trial in TNBC. Antitumor activity is seen in the neoadjuvant setting at the end of the trial, but further examination is needed [99].

### 2.3. Wnt Signaling

Wnt is a family of proteins with essential roles in numerous routes in the human body such as embryonic development and specification of cell identity [100]. Abnormality and overactivation in Wnt signaling are associated with several solid cancers like ovarian, colorectal, and breast cancers [101]. Overactivation of Wnt has been seen in more than 90% of metaplastic breast cancers. Previous studies revealed that expression of Wnt1 in mammary cells leads to increment in stem cell regeneration, resistance against apoptosis, and incapability of senescence [102]. In addition, previous *in vitro* studies detected involvement of the Wnt signaling pathway in resistance against recent oncological medicines via regulation of progenitor cell populations [103]. Downregulation of Wnt inhibitor Dickkopf 1 (DKK1) in a cell line corroborated the relevance of Wnt regulation with metastatic development in breast cancers [104]. Literature clearly demonstrates that there is a strong correlation between impairment of the Wnt signaling pathway and cancers and metastasis in human breasts. Earlier reports revealed that prohibition of the Wnt pathway favorably decreases the stem-like activity of metastatic cancer cells obtained from patients which lightens the potential of targeting Wnt for cancer therapy [105]. There is no specific enzyme as a putative target in the Wnt pathway; consequently, specifically targeting Wnt as potential therapy is wrought with complexities. Moreover, the Wnt pathway is a highly complex network with numerous functions [106].

### 2.4. Sonic Hedgehog (SHH) Signaling

The Sonic Hedgehog (SHH) signaling pathway has important role in cell growth and differentiation in the embryo. It is also known that aberrant activation of SHH leads to tumorigenesis in some organs such as the prostate, breast, and several others [107]. Following the activation of this pathway, several molecules bind the transmembrane receptor known as Patched1 (PTCH1) and induce variation in conformation structurally which mediates activation of the GLI family. GLI family has three members, GLI1, GLI2, and GLI3, which have a critical role in the regulation of Hedgehog targeted genes [108]. Hedgehog signaling induces epithelial mesenchymal transition (EMT) in the embryo and also has an important part in cancer metastasis [109] in which epithelial stromal interplay improves the invasiveness of breast cancer [110]. Disorder of the PTCH1 or GLI results in serious defects in ductal morphogenesis and may lead to human breast cancer [111, 112]. mRNAs of SHH, PTCH1, and GLI1 are highly expressed in breast tumors, inspiring the hypothesis that the SHH pathway may help predict postoperative relapse in breast cancer patients [113].

### 2.5. Breast Tumor Kinase (BRK) Pathway

Breast tumor kinase (BRK) is a nonreceptor tyrosine kinase that is overexpressed in more than 85% of malign breast cancers; however, it is significantly low or undetectable in the normal mammary glands or benign-type lesions [114]. Moreover, BRK overexpression has been shown in other cancer types such as metastatic skin cancer, colon cancer, lymphoma, and prostate cancer. In xenograft models, BRK enhanced the formation of tumors [115]. Even though previous studies strongly suggested that BRK plays a significant role in breast tumorigenesis, the cellular roles of BRK are still not fully illuminated. BRK is associated with various other signals, which are effective in breast cancers. BRK activates EGFR tyrosine kinase signals and leads to upregulation of cell growth and migration in breast cancer [116]. Interaction of BRK and EGFR also activates other signaling molecules like mitogen-activated protein kinase (MAPK) [117]. Previous studies also showed that BRK overexpression is highest in cancers which also overexpressed HER-2 and HER-4 [118]. Artificial induction of BRK in BRK-negative breast cancer cells leads to elevation of anchorage-independent growth which implies that BRK may increase the rate of metastasis [119]. Taken together, these findings suggest that BRK is an important marker and a target for novel treatments against human breast cancer.

### 2.6. HER Signaling

EGFRs are members of the tyrosine kinase receptors family and there are four members: EGFR (HER-1/ErbB1), HER-2 (ErbB2), HER-3 (ErbB3), and HER-4 (ErbB) [120]. HER-2 is considered as the most important among others since more than 30% of various breast cancers demonstrate overexpression. Thus, it is considered as a marker for tumor cell proliferation and cancer development [121]. Levels of HER-2 domain in the extracellular matrix (ECD) are found enhanced in patients with HER-2 negative tumors [122]. In addition, amplified HER-2 expression is related to higher metastatic potential and resistance to chemical pharmaceutical agents such as tamoxifen [123]. Moreover, this phenomenon also suggests that HER-2 might play an important role in the origination and advancement of human breast tumors [124]. Inhibition of HER-2 is considered as a significant therapeutic aim for human breast cancers [125]. In addition to treatment, targeting HER-2 is a useful method for diagnosis. Immunohistochemical detection (IHC), silver enhanced in situ hybridization (SISH), fluorescent in situ hybridization (FISH), and chromogenic in situ hybridization (CISH) are some accredited tools that are used in modern diagnostic implementations [122].

## 3. Signaling Pathways and Hormone Inhibitors in Breast Cancer

### 3.1. Cyclin-Dependent Kinase (CDK) Inhibitors in Breast Cancer

Cyclin-dependent kinases (CDKs) are a family of serine-threonine kinases. Since more than 2000 protein kinases have a role in the regulation of cell functions, CDKs are the most studied group due to their primary role in cell proliferation, transcription, and apoptosis [126]. CDKs are essential parts of cell cycle progression [127]. CDK/cyclin complexes are commonly decontrolled and thus overexpressed [128]. Therefore, CDK inhibitors (CDKI) have shown activity against several cancers such as breast cancer [129]. CDKIs are inhibitors of CDK proteins [106]. There are several clinical trials ongoing on CDKIs [130] in cancer therapy. A high majority of them target several CDK types. Selective CDK inhibitors are superior to nonselective counterparts since fewer adverse and toxic effects were observed for selective ones [131]. Clarification of clinically applicable potential inhibition mechanisms will be helpful in the discovery of novel alternatives for curing metastatic breast cancer [132].

### 3.2. Therapeutic Implications of Notch Inhibitors

Elevated Notch signaling is related to several cancer types such as breast, prostate, colorectal, to name a few. There are four known Notch receptors in mammals; Notch 1-4 and five ligands; Jagged-1 (JAG1), Jagged-2 (JAG2), Delta-like ligand 1 (DLL1), Delta-like ligand 3 (DLL3), and Delta-like ligand 4 (DLL4) [133]. The binding of the Notch ligand to the Notch receptor is the starting point of Notch signaling activation, and it is carried on by endocytosis of the extracellular part of the receptor notch [134]. Two types of enzymes are active in this process: ADAM group of proteinases which are a member of metalloproteinase family and *γ*-secretase complex [135]. Notch signaling regulates angiogenesis in tumors in triple-negative breast cancer [136]. Interaction with tumor microenvironment and Notch-mediated signaling in metastasis of breast cancer was shown in previous studies [137]. *γ*-Secretase inhibitors (GSIs) are a group of potential anticancer drugs with inhibitory effects on Notch signaling [138]. Several clinical trials have been carried out in patients with distinct cancer types [106]. The discovery of novel GSIs has great potential in the future prospect of cancer treatment.

### 3.3. Inhibitors of the PI3K/Akt/mTOR Pathway

#### 3.3.1. mTOR Inhibitors: The Rapalogs

**Figure 1:**
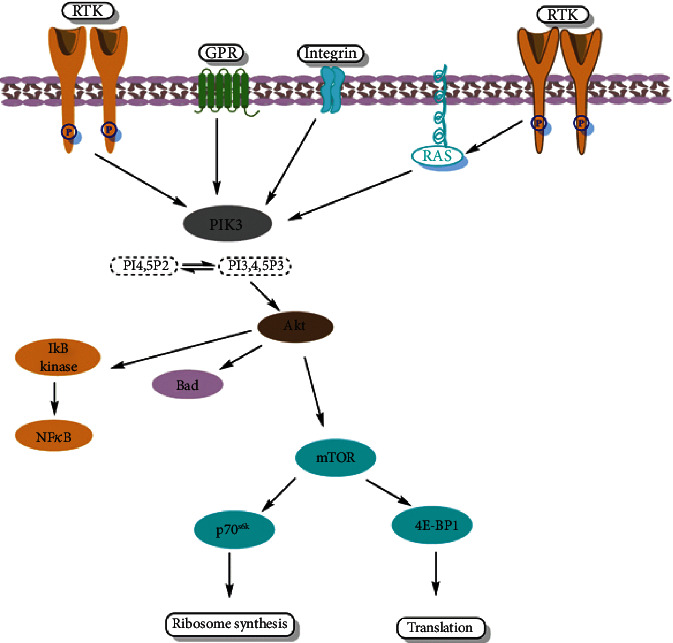
Sensitive rapamycin signaling pathways and route for inhibition of the mTOR pathway. The normal mTOR signaling pathway leads to new cell development via ribosomal machinery and through the initiation of the translation process. Hence, rapamycin inhibits the mTOR pathway that leads to inhibition of cell growth, proliferation, and cell cycle progression. RTK: receptor tyrosine kinase; GPR: G-protein coupled receptor; RAS: renin angiotensin system; PIK3: phosphoinositide-3-kinase; Akt: protein kinase-B; IkB: nuclear factor; NFkB: nuclear factor kappa light chain enhancer of activated B cells; mTOR: mammalian target of rapamycin; 4E-BP1: eukaryotic translation initiation factor; p70s6k: ribosomal protein S6 kinase.

The macrolide antibiotic, rapamycin, originates from *Streptomyces hygroscopicus* which lives in the soil on Rapa Nui Island in the 1970s. Collaboration with the mTORC1complex via binding the FKBP12-binding protein, rapamycin, and its analogs (rapalogs) suppresses downstream signaling. The inhibition of cell growth, cell cycle progression, and cell proliferation is accomplished after the inhibition of mTOR by rapamycin. Rapamycin is the first developed mTOR inhibitor (Figure 1). Due to the bioavailability problems such as poor solubility in the aqueous environment and chemical stability, rapamycin has restricted utilization in cancer therapy. Therefore, temsirolimus, everolimus, and ridaforolimus (previously deforolimus) were synthesized with improved pharmacokinetic properties. The bioactivity of rapamycin and rapalogs has been tested against endometrial and renal cancers and mantle cell lymphoma. Moreover, they have shown modest activity on solid tumors as well [139].

#### 3.3.2. Dual PI3K-mTOR Inhibitors

Phosphatidylinositol 3-kinase (PI3K) activation leads to the synthesis of phosphatidylinositol 3,4,5-triphosphate and activation of the kinases PDK1 and Akt. Subsequent to stimulation of Akt are activation and phosphorylation of the mammalian target of rapamycin (mTOR) [140]. Indeed, the PI3K/Akt/mTOR cascade is a frequently activated pathway in human cancers [141].

Dual PI3K-mTOR inhibitors do not selectively inhibit mTOR because they concurrently target the ATP-binding regions of both PI3K and mTOR with identical potency. Therefore, it is not possible to investigate these molecules in mTOR regulation or function. Because they target more than three enzymes (Akt, PI3K, and mTOR) in the PI3K signaling pathway, they are more advantageous than single-target suppressors. Suppression of mTORC1 activity by rapamycin analogs can lead to increased activation of PI3K axis due to the negative feedback loop of the mTOR-S6K-IRS1. Thus, mTOR and PI3K inhibitors may show efficacy in inhibiting the PI3K pathway reactivation [139].

Dual PI3K-mTOR inhibitors also target all PI3K, mTORC1, and mTORC2 isoforms. Although theoretically advantageous in shutting down the PI3K/Akt/mTOR pathway, possibly, it leads to enhanced toxicity. SF1126 is a prodrug of LY294002 that is conjugated to an integrin-binding component. Antitumor activity on solid tumors such as breast and prostate cancer and glioblastoma and antiangiogenic activity which is related to the reduction in HIF-1*α* levels have been demonstrated. Today, binary inhibitors are being investigated: Novartis (Basel, Switzerland), NVPBGT226 and NVP-BEZ235. NVP-BEZ235 is an imidazoquinoline derivate in oral pharmaceutical form. It binds the ATP-binding clefts of both PI3K and mTOR kinases, thus suppressing their activities. The antiproliferative effect has been shown on various cancer cell lines including HER2-overexpressing breast cancer with lapatinib and trastuzumab resistance. Moreover, it also inhibits tumor growth in PI3K-mutated xenograft models of human cancer. Researches revealed that NVP-BEZ235 definitely and individually reverses the hyperactivation of the PI3K/mTOR pathway, leading to antitumor and antiproliferative effects. Even at doses higher than 500 nM, NVP-BEZ235 totally inhibits Akt phosphorylation, irrespective of exposure duration in breast cancer cells [139]. Bhatt et al. demonstrated the efficacy of NVP-BEZ235 as well. NVP-BEZ235 was found to be more potent than molecules targeting a single member of the PI3K/Akt/mTOR pathway. NVP-BEZ235 suppressed the proliferation of principal effusion lymphoma (PEL) cell lines at low doses and even cell lines that show partial resistance to rapamycin [140].

Conjugation of ATP-competitive mTOR kinase inhibitors (TKIs) to the active regions of mTORC1 and mTORC2 thus targets mTOR signaling molecules. These molecules are known as second-generation mTOR inhibitors and bind to the ATP-binding site in the TOR kinase catalytic domain (act as ATP-competitive inhibitors), inhibiting both mTORC1 and mTORC2, downregulating mTOR signaling. They are similar to the dual PI3K/mTOR inhibitors rather than rapalogs with respect to their mechanism of action. Derepression of the negative feedback seen with rapalogs mitigates the paradoxical PI3K activation. In addition, they specifically inhibit both mTORC1 and mTORC2 without suppressing other kinases, unlike PI3K/mTOR dual inhibitors. These types of molecules are PP242, PP30, Ku-0063794, Torin1, WAY-600, WYE-354, and WYE-687.

## 4. Herbal Approach in Treatment of Breast Cancer

**Figure 2:**
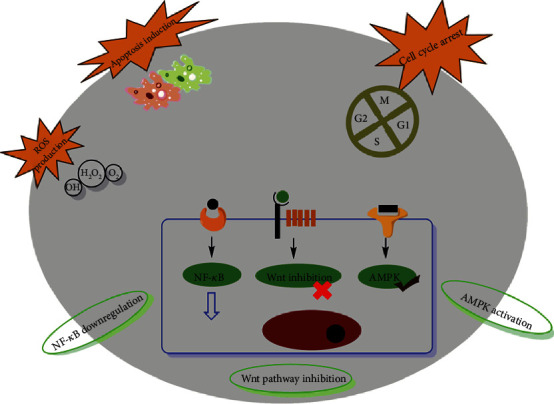
Herbal approaches in the breast cancer treatment with therapy mechanism

Natural metabolites from plants exert their effects both by directly acting on specific molecular targets like genes that have a role in altered cell cycle pathway cells and indirectly as stabilizing conjugates that influence metabolic pathways [142]. Since cancer chemotherapy and other treatments have adverse effects like nausea, mucositis, anemia, and fatigue, herbal metabolites may be preferred as an adjuvant for chemotherapy [143]. Many herbal metabolites possess anticancer properties, and they display this effect by diverse mechanisms [144–147]. Natural compounds are able to modify epigenetic events and reverse the epigenetic variations prior to cancer evolution [148, 149]. Phytochemicals may exert their activities in epigenetic modulations through cell cycle arrest, leading to apoptosis and reactivation of tumor inhibitor genes via targeting specific key transcription factors, growth factor-mediated pathways, and kinases (Figure 2) [150, 151].

In comparison with synthetic chemotherapeutics, natural products are used in breast cancer therapy since they have lower adverse effects and toxicity [152]. Natural metabolites with similar structure to estrogen have the ability to interfere aromatase expression by suppressing aromatase activity [153]. Moreover, phytochemicals also have actions on the arachidonic acid (AA) pathway, as well as metabolic enzymes phospholipase A2s (PLA2s), cyclooxygenases (COXs), and lipoxygenases (LOXs) [154]. The AA pathway has a significant role in the inflammation tumorigenesis [155, 156]. There is an association between high levels of COX-2 and lower invasiveness, prognosis, and density of breast cancer cells [157]. A positive correlation between the expression levels of COX-2 and distant metastases in breast cancer has also been documented [158]. Knocking down COX-2 reduces the metastatic appearances of breast cancer cells in mice [159]. *In vitro* and *in vivo* studies showed that phytochemicals prevent the conversion of healthy normal cells to premalignant cells and premalignant cells to malignant cancerous cells via xenobiotic biotransformation phase alteration, promotion of a more differentiated phenotype in target cells, and prevention of health cells from oxidative stress [160–162].

### 4.1. Natural Compounds as Therapeutics

#### 4.1.1. 3,3′-Diindolylmethane

**Figure 3:**
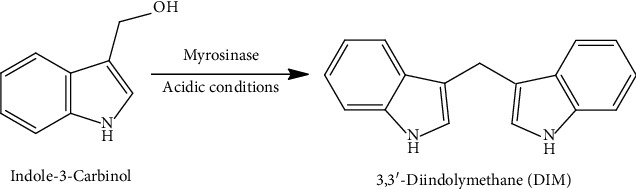
Formation of 3,3′-diindolylmethane (DIM) from indole-3-carbinol (I3C).

3,3′-Diindolylmethane (DIM) is a natural metabolite that is frequently obtained from Cruciferae plants such as cabbage, cauliflower, and broccoli. In acidic conditions, especially in the gastrointestinal system, indole3-carbinol (I3C) in plants converted to DIM (Figure 3).

By binding the aryl hydrocarbon receptor in human breast cancer cell, DIM blocks COX-2 expression. Oxidative stress leads to phosphorylation of Brca1 by DIM stimulation and plays protective roles. DIM blocks the expression of angiogenesis-expressing genes including surviving and hypoxia-inducible factor-1. DIM along with Herceptin downregulates Akt and NF-kB p65 and thus diminishes the expression of FoxM1 in HER-2/neu-expressing breast cancer cells. Correspondingly, in the case of Taxotere, DIM targets FoxM1. In randomized placebo-controlled clinic studies, DIM has been shown to increase the sensitivity of tamoxifen and positive effects on estrogen metabolism. Moreover, DIM sensitized 𝛾-radiation and induced apoptosis via cell cycle arrest at the G2/M phase and augmented ROS levels. DIM also upregulates the expression of CYP19 in MDA-MB-231 cells and diminishes aromatase expression in MCF-7 cells by acting as an aromatase inhibitor. Tumor inhibition in rodent models was similarly demonstrated by DIM.

#### 4.1.2. Curcumin

**Figure 4:**
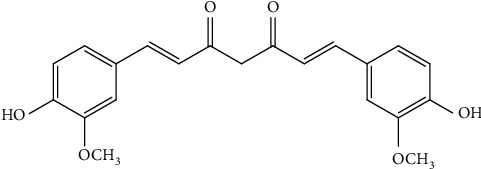
Chemical structure of curcumin.

Curcumin (1,6-heptadien-3,5-dione-1,7-bis(4-hydroxy-3-methoxyphenyl)-(1E, 6E)) is an active principle of *Curcuma longa* L. (turmeric, Zingiberaceae) a polyphenolic metabolite (Figure 4). It is a well-known medicinal ingredient against various diseases, most importantly anti-breast cancer properties. This metabolite shows its anticancer activity by modulating various pathways. Curcumin induces apoptosis via modulating the expression of apoptosis-related genes and proteins. Lately, it was revealed that curcumin might exaggerate apoptosis in breast cancer cells via inducing the concentration of p53 in turn enhancing Bax expression thus increasing Bax/Bcl-2 ratio. Therefore, it leads to programmed cell death. Curcumin also downregulates NF-𝜅B expression which is important in cell proliferation. Thus, alleviated expression of NF-𝜅B manages antiproliferative activity on BT-483 and MDA-MB-231 cells. An additional study showed that curcumin alleviates the protein expression of urokinase-type plasminogen activator via NF-𝜅B activation thus blocking the adhesion and invasive nature of MCF-7 cells.

Cancer stem cells are also affected by curcumin. Wnt signaling in MCF7 cells is suppressed by curcumin that is dysregulated in breast cancer patients. Via inhibition of this pathway, curcumin is suggested as a potent anti-breast cancer chemotherapeutic [163].

Curcumin is a possible histone modulator, and it regulates the enzymatic activity of HDACs and HATs. It is known that curcumin inhibits class I HDCA expression and upregulates the expression of several miRNAs associated with carcinogenesis to lower the expression of Bcl-2 [163].

Analogous to several other plant metabolites, curcumin increases the activity of other chemotherapeutics such as paclitaxel. Via inhibiting NF-𝜅B expression, curcumin increases the activity of paclitaxel. This synergism decreases breast cancer growth in MDAMB-231 (ER−/PR−) cells and downregulates MMP-9 expression along with tumor size and tumor cell proliferation and with the increased apoptosis rate [163].

Although many potential effects of curcumin on breast cancer have been demonstrated, low bioavailability limits its clinical utilization.

#### 4.1.3. Epigallocatechin Gallate

**Figure 5:**
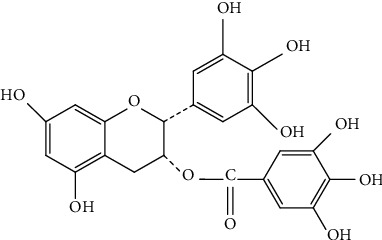
Chemical structure of (−)-epigallocatechin-3-gallate.

Epigallocatechin gallate (EGCG) ([(2R,3R)-5,7-dihydroxy-2-(3,4,5-trihydroxy phenyl) chroman-3-yl] 3,4,5-trihydroxybenzoate) (Figure 5) is among the most common phenolic catechins in nature, found in green tea, and is renowned for its positive health effects.

EGCG have epigenetic effects on cancerous cells, by demethylation or by inhibition of methylation of tumor suppressant gene promoters. Combination therapy with class I HDAC inhibitor, trichostatin A (TSA), and EGCG promotes ER*α* expression in ER*α*-negative MDA-MB-231 breast cancer cells via histone methylation modulation and acetylation at gene promoter. Breast cancer cell treatment with EGCG might activate the expression of the repressed TIMP-3 gene by epigenetics. Modulation of epigenetic mechanisms including EZH2 and class I HDACs independent of the promoter DNA methylation controls the TIMP-3 gene. EGCG treatment affects the protein content of class IHDACs and EZH2 by reducing their amount significantly. EGCG prevented the spread of the estrogen-sensitive MCF-7 breast cancer cell line as well as the binding of ER5-007 and Er*β*. EGCG induces apoptosis by ER-independent acts to inhibit aryl hydrocarbon-(AhR-) regulated genes. The antiproliferative effects of EGCG are mediated by blocking the ER𝛽-specific inhibitor PHTPP. Moreover, EGCG induces apoptosis in ER-negative MDA-MB-231 and MDA-MB-468 cell lines also alter the EGFR potential which is associated with tumor growth; EGCG increases protein expression of p21 and p27 along with the increased expression of proapoptotic genes, caspase-3, caspase-8, caspase-9, and TP53. Furthermore, EGCG inhibits the arachidonic acid pathway via affecting COX-2 expression by minimizing the activity of the COX-2 promoter through inhibition of NF-𝜅B activation. However, EGCG does not affect aromatase activity. In clinical studies in breast cancer patients, EGCG promoted the sensitivity to radiation and displayed protective activity against adverse effects of chemo- and radiotherapy. The bioavailability of 5-fluorouracil, doxorubicin, and tamoxifen was increased by combination therapy of EGCG. EGCG is founded to be safe and well tolerated in doses up to 1600 mg [163].

EGCG has the ability to restore tumor suppressing like retinoid X receptor alpha, leading to inhibition of breast cancer via binding to many high-affinity target proteins, for instance, 70 kDa zeta-associated protein (Zap-70). In docking studies, it was shown that mTOR and PI3K signaling binds to the PI3K kinase active site, showing ATP-competitive effect in several cancers including MDA-MB-231 [164].

#### 4.1.4. Biochanin A

Biochanin A is a secondary metabolite in isoflavone structure and obtained from *Trifolium pratense* L. (red clover, Fabaceae) with anticancer properties. It blocked the activity of the aromatase as well as cell growth on MCF-7 cells transfected with the CYP19 gene. Moreover, in ER-negative breast cancer cells, it suppresses aromatase enzyme activity and reduces mRNA expression. When compared with genistein, biochanin A is better tolerated and leads to positive expression of tumor-inhibiting genes in MCF 12A, MCF7, and HMEC (ER-positive) cell lines. A xenograft mouse model demonstrated that biochanin A is very potent to diminish the growth of estrogen-dependent MCF-7 tumors [163].

#### 4.1.5. Genistein

**Figure 6:**
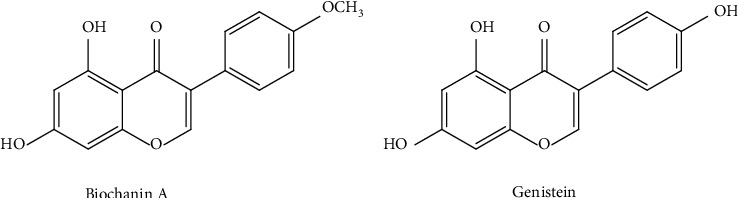
Chemical structure of biochanin A and genistein.

Genistein is a metabolite of biochanin A, with an analogous isoflavonoid structure (Figure 6). Like other soy isoflavones, genistein controls COX-2 expression and antagonize AA to control PGE2. Genistein also prevents inflammation by suppressing sPLA2 activity and reduces COX-2 expression in MCF-7 breast cancer cells thus preventing breast carcinogenesis.

Furthermore, the transcriptional activity of NF-𝜅B in MCF10A human breast epithelial cells and TPA-induced COX-2 expression are blocked by inhibiting ERK-mediated phosphorylation of p65. Because of the structural similarity with estradiol (E2), genistein activates ER*α* and ER𝛽. Genistein induces apoptosis in breast cancer cell lines via upregulating Bax and p21WAF1 proteins in MDA-MB-231 cell lines. In addition, genistein induces apoptosis via controlling calpain-caspase-7 and protein kinase activation cascade and apoptosis signaling kinase 1-p38 mitogen-activated protein kinase activation cascades. Moreover, apoptotic mechanism might be related to the cellular Ca^2+^ regulatory activity. Genistein also inhibits cell proliferation, as it inactivates the IGF-1R-PI3K/Akt pathway and reduces Bcl-2/Bax mRNA and protein expressions. Another pathway that genistein targets is the ATM/Chk2/Cdc25C/Cdc2 checkpoint pathway. It activates this pathway and enhances G2/M arrest thus increasing the radiosensitivity of both ER+ and ER− breast cancer cells by an apoptosis pathway mediated by mitochondria.

Genistein has been shown to be a potent chemotherapeutic in the initial phases of breast tumorigenesis through epigenetic regulations. It regulates p16 and p21 by playing a role in histone variations. Consequently, it improves *ERα* expression, which in turn boosts the sensitivity of TAM-related antiestrogen therapeutic. Genistein modulates the regulation of Brca1 and Brca2 mRNA expressions in elder ovariectomized rats [163].

#### 4.1.6. Lycopene

**Figure 7:**
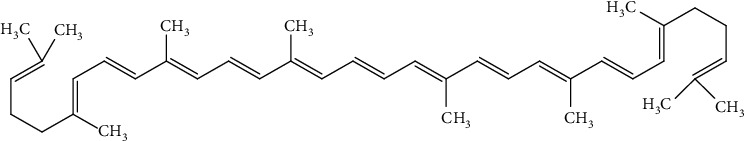
Chemical structure of lycopene.

Lycopene, a carotenoid that is an important part of the human diet, is found in red or yellow fruits particularly in tomatoes, carrots, watermelon, and papayas (Figure 7) [165]. Lycopene is a powerful antioxidant, and its effects on cell cycle, proliferation, and apoptosis were reported on different breast cancer models [166–168].

The antiproliferative and cytotoxic activities of lycopene were reported in various cell lines *in vitro*. Lycopene was shown to significantly decrease the viability of MCF-7 cells and promoted cell cycle arrest and inhibited cellular proliferation [167–169]. Lycopene treatment in MCF-7 cells decreased cellular proliferation and increased apoptosis of MCF-7 cells in a dose- and duration-dependent manner. The mechanism appears to be related to regulation of p53 and Bax mRNA expression in MCF-7 cells [168]. Furthermore, lycopene inhibited cellular growth and cell cycle progression in T-47D and MCF-7 cell lines secondary to reduction in cyclin D levels and inhibition of pRb phosphorylation. The retention of p27 in cyclin E–CDK2 complex was also involved, leading to inhibition of G1 CDK activity. Lycopene also inhibited cell cycle progression induced by insulin-like growth factor- (IGF-) 1 and decreased cyclin D1 levels [170, 171].

Growth stimulation by IGF-I was significantly decreased by lycopene in MCF7 cells. The effects were not reported to be associated with necrotic or apoptotic cell death. The binding capacity of the AP-1 transcription complex and IGF-I activation of tyrosine phosphorylation of insulin receptor substrate 1 were reduced by lycopene application, suggesting that the effects were due to the interference with IGF-I receptor signaling and cell cycle progression [172].

Lycopene treatment also reduced the viability of MDA-MB-231 breast cancer cells; however, the antiproliferative activities were slight and migration has not been affected [173]. On the other hand, lycopene inhibited cell proliferation, increased apoptosis, and arrested the cell cycle in different phases in MCF-7, MDA-MB-231, and MDA-MB-235 cells [169]. Moreover, lycopene inhibited invasion, migration, and proliferation of MDA-MB-231 and H-Ras MCF10A cells. ERK and Akt activation in H-Ras MCF10A cells was also inhibited, suggesting that these signaling pathways might play roles in the mechanism of the observed effects [174]. The inhibitory effects of lycopene treatment on the cellular growth of MDA-MB-231 cells were explained by the inhibition of the phosphorylation of inhibitor of kappa B (I*κ*B) protein, the transcriptional activity of NF-*κ*B, and the TNF-induced nuclear translocation of NF-*κ*B p65 subunit at concentrations that are relevant *in vivo* [175]. Downregulatory effects of lycopene on Skp2 were also reported in MDA-MB-231 and BT474 cells [176]. A study compared the effect and the activity mechanism of lycopene on three subtypes of human breast cancer cell lines with different hormone receptors and HER2 status. MCF-7 cell line (ER/PR-positive), SK-BR-3 cell line (HER2-positive), and MDA-MB-468 cell lines (triple-negative) were treated with lycopene. In all cell lines, lycopene arrested the cell cycle at the G0/G1 phase and induced ERK1/2 activation thus exhibiting antiproliferative activity. Lycopene treatment also induced cyclin D1 suppression and p21 upregulation. On the other hand, lycopene inhibited Akt and mTOR phosphorylations and subsequently, proapoptotic Bax was upregulated which was not accompanied by any effect on antiapoptotic Bcl-xL in triple-negative cells [177].

In a study where carcinogenic (MCF-7) and noncarcinogenic (MCF-10) cells were compared, MCF-7 cell viability was inhibited by lycopene treatment while noncarcinogenic cells were unaffected. The data suggested that an oncogene might be present as a target to observe the effect [178]. The mRNA levels of oncosuppressor genes BRCA1 and BRCA2 were increased in MCF-7 and HBL-100 cells (estrogen receptor- (ER-) positive) by lycopene treatment. However, the mRNA levels of these genes are decreased or did not have an effect in MDA-MB-231 and MCF-10a cells (ER-negative cell lines) [179]. Lycopene was also reported to upregulate the expression of the GSTP1 tumor suppressor gene in breast cancer cells. Lycopene also demethylated GSTP1 promoter in MDA-MB-468 cells [180]. Lycopene was shown to have a regulatory effect on apoptosis, as well as cell cycle and DNA repair mechanisms according to the receptor cell status of estrogen and retinoic acid. Moreover, a whole-genome microarray hybridization technique revealed that lycopene exposure leads to differential gene expression in MCF-7 and MDA-MB-231 cell lines. Modified gene expression was reported in numerous molecular pathways, including cell cycle and communication, apoptosis, and MAPK [181, 182].

#### 4.1.7. Shikonin

**Figure 8:**
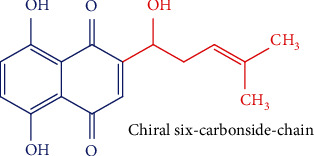
Chemical structure of shikonin.

Shikonin is a type of 1,4-naphthoquinone derivative with benzene moiety linearly fused with a completely conjugated cyclic diketone, in which the carbonyl groups are arranged in paraorientation, linked to a chiral six-carbonside chain (Figure 8). Shikonin is the major compound of *Lithospermum erythrorhizon* Siebold & Zucc. roots from the Boraginaceae family [183].

The anticancer activities of shikonin were shown *in vitro* and *in vivo*, and several studies were also conducted on molecular signaling pathways related to cancer development [184–187]. One of the earliest researches indicates cytotoxic effects of shikonin in human breast cancer cells (MCF-7) via apoptotic processes [188]. Shikonin was shown to exert an inhibitory effect on MCF-7 cellular proliferation via reducing tumor-derived exosomal miR-128. It has been suggested that shikonin suppressed the growth of MCF-7 cells by exosome release inhibition. It was also shown that Bax expression in recipient MCF-7 cells was suppressed by the exosomal miR-128 [186]. Shikonin inhibited the growth and cellular proliferation of SKBR-3 and MDA-MB-231 cells along with MCF-7 cells. The cellular proliferation is inhibited in MDA-MB-231 via arresting the cell cycle at the G1 phase. Shikonin was also shown to induce apoptosis in MDA-MB-231 cells. RNA-seq transcriptome analysis revealed that shikonin induced DUSP1 and DUSP2 expressions. The JNK and p38 MAPK pathways were also inhibited by the shikonin treatment which resulted in apoptosis and cell cycle arrest [187]. Moreover, shikonin suppressed MDA-MB-231 and 4T1 cell viability and terminated the capacity of cell migration and invasion. The molecule significantly upregulated E-cadherin and downregulated N-cadherin, Snail, and vimentin. By reversing the epithelial-to-mesenchymal transition, shikonin was shown to inhibit metastasis the mechanism of which was suggested to be via suppression of *β*-catenin signaling by glycogen synthase kinase 3*β* [189]. The suppression of migration and invasion of MCF-7 cells by shikonin treatment is reported to be through the modulation of matrix metalloproteinase- (MMP-) 9 [190]. Apoptosis was induced by shikonin treatment in 4T1 murine mammary cancer cells as well as MDA MB 231 cells. It is revealed that shikonin induced apoptosis in breast cancer cells in a caspase-dependent manner and regulated by the p38 pathway instead of the JNK signaling pathway [191]. Shikonin's activity on apoptosis and necroptosis, along with the underlying mechanism, was investigated by Shahsavari et al. [192–194]. Shikonin was shown to induce caspase-3 dependent apoptosis. The ROS production in the mitochondria of T-47D cells was also stimulated which resulted in necroptosis or apoptosis [192]. In a subsequent paper, it was reported that shikonin-mediated cell death occurred via RIP1K-RIP3K-induced necroptosis in MDA-MB-468 cells [193]. The mechanism of necroptosis and apoptosis mediated by RIPK1-RIPK3 was also studied in MCF-7 cells. Shikonin was found to induce necroptosis and apoptosis, and RIPK1 and RIPK3 expressions were increased. The percentage of the cells in sub-G1 and later stages of the cell cycle was also increased [194].

Several studies also addressed the effects of shikonin on estrogen receptor-dependent pathways. A study investigated the antiestrogen effects of shikonin in MCF-7, T47D, and MDA-MB-231 cells. Shikonin inhibited tumor cell growth in ERalpha-positive cells but not ER-alpha-negative cells. Shikonin cotreatment inhibited estrogen-dependent cell growth. A potential mechanism by which shikonin inhibits estrogen action was proposed to be a decrease in ER-alpha protein levels associated with an increase in ubiquitinated ER-alpha for degradation. The recruitment of ER-alpha at the estrogen-dependent gene promoters was also inhibited by shikonin treatment by which the gene expression is suppressed [195]. NRF2-dependent enzymes are inhibited by the estrogen-receptor signaling pathway. Shikonin has been reported to contribute to breast cancer prevention by reversing the inhibitory effects of estrogen on this pathway [196]. The mRNA and enzymatic activity of steroid sulphatase which is crucial for the biosynthesis regulation of estrogen in breast cancer was downregulated by shikonin treatment [197]. The proliferation of MCF-7 cells was inhibited by shikonin treatment possibly by arresting the cell cycle at the G0/G1 phase and apoptosis. Shikonin exerted antitumor effects on SK-BR-3 as well as MCF-7 cells. The effects were reported to be related to EGFR/p-ERK downregulation via estrogen receptor (ER) *α* and G protein coupled estrogen receptor (GPER) inhibition [198].

Shikonin was shown to inhibit the activation of STAT3 (signal transducer and activator of transcription 3) which is hyperactivated in tumor cells as well as the activations of FAK and Src [185]. Shikonin was reported to inhibit preadipocyte signaling through the inhibition of nearby DCIS. Secretion of exosomes with high levels of miR-140 by shikonin treatment affected adjacent DCIS cells by targeting SOX9 signaling [199].

#### 4.1.8. Sulforaphane

**Figure 9:**
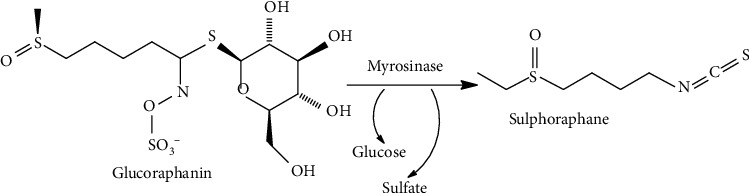
Glucoraphanin is hydrolyzed by myrosinase to yield glucose, sulfate, and sulforaphane.

Glucosinolates are important phytoconstituents of the Brassicaceae family which are hydrolyzed into active isothiocyanates. Sulforaphane is an isothiocyanate which is the hydrolyzation product of glucoraphanin (Figure 9) [200]. The effects of sulforaphane on *in vitro* and *in vivo* models of breast cancer and its synergistic effects with other treatment options were studied [200–204].

Sulforaphane induced apoptosis in MCF-7 cells through downregulation of Bcl-2, and the efficacy of gemcitabine might be potentiated by combinational treatment of sulforaphane with gemcitabine [201]. Cell invasion of MCF-7 cells was also decreased by sulforaphane treatment, accompanied by reduction of TPA-induced MMP-9 which is responsible for the degradation of the extracellular matrix and involves in cancer cell invasion. TPA-stimulated NF-*κ*B-binding activity was also inhibited by sulforaphane treatment through inhibiting phosphorylation of I*κ*B, but not MAPK or AP-1-binding activity [202]. Sulforaphane inhibited cellular proliferation in MCF-7 cells and decreased ER-alpha protein and progesterone receptor expressions. The mechanism by which sulforaphane inhibited the expression of ER-alpha protein was partially revealed to be by ER-alpha mRNA transcription and by a proteasome-mediated degradation process [205]. The metabolic changes in MCF-7 cells treated with estradiol (E2) and/or sulforaphane were also investigated. E2 and sulforaphane treatment-induced metabolites were found to be associated with differences in energy metabolism, glycolysis, amino acid, purine, and folic acid metabolism. The epigenetic status of MCF-7 cells appeared to be affected by E2 and sulforaphane via the folate pathway [206]. Moreover, sulforaphane was chemopreventive against ER-positive and cyclooxygenase-induced breast cancers. Sulforaphane treatment also reduced the proliferation in MCF-7 cells. 12-TPA-induced cyclooxygenase-2 expression in M13SV1 cells (immortalized human breast luminal epithelial cells) was inhibited. In addition, the upregulating effects on p38 and activation of caspase-7 in MCF-7 cells might explain its role in cell survival and apoptosis [207]. Sulforaphane inhibited the migration of cells and induced apoptosis in MDA-MB-231 cells. Moreover, a reduction in the expression of genes involved in epithelial mesenchymal transition such as ZEB1, fibronectin, and claudin-1 was detected. The inhibition of a key mediator of the Wnt pathway, *β*-catenin, was involved partly in the apoptotic and antimetastatic effects [208]. In a study designed to investigate the effects on the invasive behavior of MDA-MB-231 cells, sulforaphane was found to downregulate PBR and vimentin expression along with MMP7 and MMP14 mRNA. Transcription factors that regulate EMT and self-renewal of undifferentiated embryonic stem cells such as Twist1 and POU5F1 were also downregulated. The production of proinflammatory cytokines (IL-1*β*, IL-4, IL-6, TNF*α*, and IFN-gamma) and growth factors involved in angiogenesis such as PDGF and VEGF were also reduced. The data suggested that the effects of sulforaphane are associated with the reversal of the parameters connected with extracellular proteolysis, EMT, and matrix degradation and with the reducement of the proangiogenic growth factors and proinflammatory cytokines [209]. The retardation in growth and inducement of cell death of MDA-MB-231 cells by sulforaphane were accompanied by S and G2/M cell cycle arrest. This effect was associated with increased levels of p27KIP1 and p21WAF1 as well as the decreased levels of cyclin A, cyclin B1, and CDC2 levels. It was found that cell death was due to apoptosis as caspase-3 levels were increased concomitant with lower levels of BCL-2 [210]. Sulforaphane has been reported to induce cell type-specific apoptosis in human breast cancer cell lines. The molecule inhibited the growth of cell lines and induced a G2-M block which is observed with an increase in cyclin B1 protein expression. HDAC activity is significantly inhibited by sulforaphane treatment, particularly in the ER-negative cell lines, and the protein expressions of ER, HER-2, and EGFR were decreased [211]. The growth of phenotypically different cell lines (MCF-7, SKBR-3, MDA MB 231, and MDA MB 468) was inhibited with alterations of the PI3K-Akt-mTOR-S6K1 pathway [212]. Using MCF-7 and MDA-MB-231 cells, sulforaphane was reported to be a potent inducer of apoptosis owing to Heat Shock Protein (HSP) modulation. A downregulation in the expressions of HSP70, 90, and HSF1 was observed concomitant with p21 upregulation. Upregulation of apoptotic proteins Bax, Bad, and Apaf-1 was followed by downregulation of Bcl-2. Alteration in Bcl-2 Bax ratio caused cytochrome c release from mitochondria and caspases 3 and 9 activations [213].

Sulforaphane inhibited the proliferation and survival of ZR-75-1 breast ductal carcinoma cells, regardless of necrosis or apoptosis. However, sulforaphane induced accumulation of G1 phase cell population and induced cell cycle arrest through CDK4 downregulation. SERTAD1 and CCND2 expressions were also decreased significantly [214].

Sulforaphane inhibited the proliferation of TNBC cells as well as suppressed mammary tumor development in an animal model of TNBC. The suggested mechanism was reported to be by targeting cancer stem-like cell population. Gene analysis demonstrated that the molecule decreased the expression of numerous stem cell markers such as cancer-specific CR1 and CR3 (a homologue of CR1), Nanog, aldehyde dehydrogenase 1A1 (ALDH1A1), Notch4, and Wnt3 [203]. Sulforaphane is reported to inhibit breast CSCs *in vitro* and *in vivo*, and a possible mechanism is suggested to be the downregulation of Wnt/beta-catenin self-renewal pathway. A stabilized formulation of sulforaphane within an alpha-cyclodextrin complex was prepared used in combination with endocrine therapies. The treatment resulted in the prevention of breast CSC enrichment in patient samples and *in vivo*. The mechanism underlying this effect was reported to be probably due to its direct STAT3 targeting [215].

Other pathways were also studied to explain their role in the mechanism of anticancer effects of sulforaphane. Sulforaphane was shown to be an epigenetic modulator in breast cancer due to induction of cell cycle arrest and senescence. Genotoxicity, nitrooxidative stress, and diminished AKT signaling were observed as well as an elevation of the levels of p21 and p27. DNA hypomethylation and alleviated levels of DNA methyl transferases (DNMT1, DNMT3B) were reported to mediate these effects. Sulforaphane also affected microRNA profiles. In three breast cancer cells, sulforaphane significantly decreased the levels of miR-23b, miR-92b, miR-381, and miR-382 [216]. Using a competition-based, quantitative chemical proteomics method, various sulforaphane binders including KEAP1, MIF, and NF-*κ*B subunits p65 and p52 were identified in breast cancer cells along with other apoptosis signaling targets such as DFFA, BID, and ROCK1 and proteins such as STAT1 and STAT3 [217]. Sulforaphane inhibited human telomerase reverse transcriptase (hTERT) expression, in both MDA-MB-231 and MCF-7 cells. The effects were suggested to be due to epigenetic pathways because DNA methyltransferases (DNMT1 and DNMT3a) were similarly decreased [218]. It was also reported to inhibit mitosis by microtubule stabilization [219]. In addition, sulforaphane is reported to be a nucleolar stress inducer that leads to the inhibition of breast cancer cell proliferation. Sulforaphane elevated superoxide levels and protein carboxylation that cause unbalanced lamin B1/lamin A/C ratio accompanied with alterations of organization of nuclear lamina and abnormal morphology of the nucleus. Nucleoplasmic translocation of RRN3 and inhibition of rRNA synthesis are observed in nucleolar stress response [220]. The link between inflammation and cancer as manifesting itself in an aberrant production of COX-2 prompted researchers to investigate the molecular mechanism of COX-2 inhibition on TPA-induced human mammary epithelial (MCF-10A) cells. Sulforaphane is reported to inhibit NF-*κ*B activation and COX-2 expression induced by TPA in MCF-10A cells. The signaling pathways including ERK1/2-IKK*α* and NAK-IKK*β* were also blocked suggesting that these pathways might be responsible for the observed effects [221].

The *in vitro* and *in vivo* mammary cancer-suppressive effects of sulforaphane were also demonstrated by Jackson and Singletary using BALB/c mouse mammary carcinoma cell line F3II and BALB/c mice injected *s.c.* with F3II. The mechanism involved the inhibition of tubulin polymerization and early M-phase block accompanied by CDC2 kinase activation [222]. Cao et al. reported that the antineoplastic effect of the molecule is regulated by the HDAC5-LSD1 axis using a combination of *in vivo* and *in vitro* methods. In this study, HDAC5 transcription was downregulated by sulforaphane. The LSD1 ubiquitination and degradation were facilitated in an HDAC5-dependent manner. The cross-talk between HDAC5 and LSD1 is reported to be crucial for the antitumor efficacy of sulforaphane. Breast cancer growth was blocked by HDAC5-LSD1 axis inhibition. The combination of the treatment with LSD1 inhibitor resulted with improved therapeutic effect of sulforaphane [223]. Sulforaphane was reported to inhibit cell proliferation in TNBC cell lines via inducing G2/M phase arrest and apoptosis which was supported by the results of nude mouse xenograft assays *in vivo*. Sulforaphane repressed expression of cyclinB1, CDC2, and phosphorylated CDC2. Moreover, the tumor suppressor Egr1 gene was suggested to be a mediator of antiproliferative effects [224]. Cornblatt et al. investigated whether oral sulforaphane treatment reaches the mammary gland and elevates the capacity of enzymes that has antioxidant and detoxification functions in this tissue using *in vivo* and clinical methods. After treatment, elevated levels of heme oxygenase-1 (HO-1) and cytoprotective NAD(P)H: quinone oxidoreductase (NQO1) gene transcripts were measured in the rat mammary gland. After oral application of sulforaphane to eight healthy women experiencing reduction mammoplasty, sulforaphane metabolites were found in measurable amounts in breast tissue [225].

**Figure 10:**
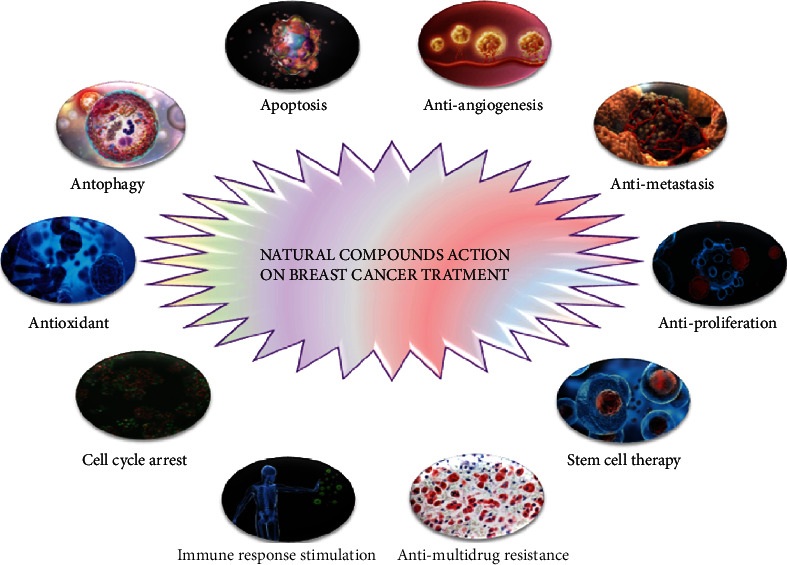
Natural compounds as therapeutics for breast cancer—routes of actions.

The synergistic effects of sulforaphane with other treatment options were also studied with a focus on the probable mechanism of action. Burnet et al. showed that treatment of paclitaxel or docetaxel induces inflammatory cytokine secretion which results in the enrichment of CSCs in TNBC cell lines. However, sulforaphane eliminates CSCs. The mechanism of action was reported to be through preventing the nuclear translocation of NF-*κ*B p65 subunit and downregulating p52. Docetaxel and sulforaphane combination exerted more decrement in primary tumor volume and decreased secondary tumor formation compared to either monotreatment *in vivo* [226]. Besides acting synergistically with doxorubicin in cancer regression, sulforaphane is found to be protective for the heart from DOX toxicity through Nrf2 activation [227]. Sulforaphane inhibited cellular growth of MDA-MB-231 and BT549 cells. The mechanistic study revealed that inducement of autophagy is probably due to the downregulation of the expression of HDAC6. As a result, phosphatase and tensin homolog (PTEN) activation was suppressed. Cotreatment of sulforaphane and doxorubicin was reported to show a synergistic inhibition on the cellular growth of TNBC cells suggesting that sulforaphane-induced autophagy sensitizes TNBC cells to doxorubicin. The combination also exerted a higher inhibitory effect on the growth of MDA-MB-231 xenografts *in vivo* relative to either treatment alone [228]. In apoptotic signaling pathways such as caspase-3, caspase-8, and caspase-9, cytochrome c was found to be activated with a combination treatment of sulforaphane with paclitaxel compared to individual treatment. Besides, the combination treatment led to a downregulation of the NF*κ*B signaling pathway and decreased the levels of Bcl-2 protein expression and phosphorylated AKT serine/threonine kinase [229]. The synergistic activity of sulforaphane and 5-fluorouracil was also reported on the inhibition of the growth of MDA-MB-231 cells through autophagy cell death and premature senescence. The results were confirmed by increased *β*-galactosidase activity and p21 protein expression accompanied by decreased cyclin B levels [230]. Chirumbolo and Bjørklund discussed these findings in a letter to the editor proposing that a possible mechanism consists of the Nrf2-KEAP1-ARE signaling pathway [231]. Clofarabine application together with sulforaphane inhibited cancer cell growth and reactivate DNA methylation silenced cyclin-dependent kinase inhibitor 2A (CDKN2A), tumor suppressor, in breast cancer cells [232]. Sulforaphane potentiated the anticancer activity of 4-hydroxytamoxifen which is reported to be mediated by downregulating Bcl-2 and surviving [233]. Sulforaphane was also reported to sensitize HER2-positive breast cancer cells to lapatinib treatment. This effect was observed with a reduction in phosphorylation of HER2, Akt, and S6 [204]. The schematic diagram of natural compounds on breast cancer treatment is shown in Figure 10.

## 5. Future Prospect of Herbal Management

Conventional treatment regimens in breast cancer have several shortcomings, such as serious side effects as well as MDR. Chemotherapy and radiotherapy frequently generate various unwanted side effects in cancer patients. In addition, MDR diminishes the success rates of the therapies. Contrarily, phytochemicals might act synergistically with several chemotherapeutics by increasing their potency. Up to date, numerous natural metabolites prove this synergistic hypothesis. For instance, 3,3′-diindolylmethane (DIM), genistein, and equol promote the efficacy of paclitaxel, doxorubicin, and tamoxifen, respectively [234–236]. Similarly, the extract of *Rosmarinus* sp. increases the efficacy of tamoxifen, trastuzumab, and paclitaxel [237]. Thus, natural compounds are promising agents in the treatment of breast cancer. Although chemotherapy is the most prominent medication strategy, it may cause chemoresistance or MDR which is a very crucial problem [238]. ATP-binding cassette (ABC) transporter action might lead to flowing chemotherapeutics out of the cells, namely, a type of resistance [239]. Thus, research has focused on natural metabolites acting against chemoresistance. Doxorubicin-resistant human breast carcinoma MCF-7 cells respond to *β*-elemene [240]. Moreover, DIM may also act as a radiosensitizer in chemoresistance and natural compounds should be tested against this pathway as well. Although triple-negative breast cancer (TNBC) is the most destructive and life-threatening subtype and treatment strategies are deficient, natural compounds showed promising results. Curcumin, resveratrol, EGCG, and carnosol are found to be effective in the treatment of TNBC via several mechanisms with lower side effects [241–244]. This review emphasizes several natural metabolites acting on mechanisms related to breast cancer. Among them, many of them exert their activity even on chemoresistant types. Altogether, natural compounds have a significant role in the prevention and treatment of breast cancer. Subsequent to the enlightening of the tumor microenvironment and pathways in breast cancer, more natural compounds with significant antitumor activity may be discovered.

## Data Availability

The data used to support the findings of this study are all included and available within the article.
